# Analysis of emergency medicine clerkship grades by identification as underrepresented in medicine (URiM) versus non‐URiM

**DOI:** 10.1002/aet2.11045

**Published:** 2025-02-12

**Authors:** Kevin Walsh, Joseph House, Elizabeth Holman, Laura R. Hopson

**Affiliations:** ^1^ Department of Emergency Medicine University of Michigan Ann Arbor Michigan USA; ^2^ Department of Emergency Medicine and Pediatrics University of Michigan Medical School Ann Arbor Michigan USA; ^3^ University of Michigan Medical School Ann Arbor Michigan USA

## Abstract

**Background:**

Previous studies identified racial differences in core clinical clerkship evaluations and components of residency applications, including the medical school performance evaluation and standard letter of evaluation. However, there are no studies that have examined grading differences in the emergency medicine (EM) clerkship. Our goal was to determine whether there are differences in EM clerkship grades and its components (National Board of Medical Examiners [NBME] exam scores and clinical assessments) between underrepresented in medicine (URiM) and non‐URiM students.

**Methods:**

This retrospective sample was drawn from University of Michigan Medical School students with graduation year (GY) 2021 or 2022 who completed the required EM clerkship. We compared overall composite scores on the EM clerkship, EM NBME exam score, and clinical assessments between URiM and non‐URiM students.

**Results:**

A total of 334 students completed an EM rotation in GY 2021 and 2022. Eleven students with “missing” race data were excluded. Fifty‐two (16.1%) identified as URiM while 271 (83.9%) identified as non‐URiM. Non‐URiM students significantly outperformed the URiM group (non‐URiM mean 81.2, URiM mean 77.6; *p* = 0.0001). There was no statistically significant difference for clinical performance (6.71 vs 6.49 *p* = 0.057). Overall clerkship grades differed, as URiM students had higher percentages of “pass” grades (32.7%) and lower percentages of “honors” grades (40.4%) than non‐URiM students (13.7%, 59.4%). When controlling for NBME shelf exam score, there were still differences in outcomes between URiM and non‐URiM students.

**Conclusions:**

There are grading differences between students who identified as URiM and non‐URiM. There is a statistically significant difference with respect to outcomes on NBME shelf exam scores, which is responsible for a portion of these differences; however, when controlled for NBME scores, there was still a difference between these two groups. This calls for a change in how students are evaluated to address equity concerns in clinical assessments.

## INTRODUCTION

Racial disparities are prevalent throughout the health care system, directly affecting both access and quality of care.^1^ Studies show that a diverse workforce can contribute to eliminating these differences, as minority providers are more likely to care for patients in underserved communities. Additionally, having a diverse health care team increases trust, improves communication, and provides better care to patients of ethnic minorities as assessed by satisfaction scores and other health outcomes.^2^

These racial differences are not limited to direct patient care, as similar differences exist in undergraduate medical education. Many studies demonstrate that White medical students have outscored their peers who identify as underrepresented in medicine (URiM) in core clinical clerkships.^3^, ^4^, ^5^, ^6^ Disparities are also present in many aspects of undergraduate medical education including standardized testing scores such as on the United States Medical Licensing Exam (USMLE), presence of more favorable comments on the medical school performance evaluation, and likelihood of election to honors societies including Alpha Omega Alpha Society.^7^, ^8^, ^9^, ^10^ These differences can have a direct impact on applicants’ success during the residency match, especially in more competitive specialties.^11^, ^12^, ^13^, ^14^, ^15^, ^16^, ^17^, ^18^, ^19^, ^20^ With these discrepancies, it can be difficult to improve the diversity of health care teams to address these health care disparities. As a result, many residency programs are now using a holistic review approach to review residency applications with the goal of increasing diversity in their respective residency programs.^20^

Emergency medicine (EM), as a specialty, is not exempt from these challenges. Racial differences exist in the heavily weighted EM standard letter of evaluation.^21^, ^22^ While there are multiple studies documenting racial differences in core clerkship grades,^3^, ^4^, ^5^ only one study included EM in their analysis.^5^ No study specifically examines differences in clerkship grades with respect to EM clerkship scores and the impact of the different grade components to investigate the source of these grading discrepancies.^5^

Our study sought to understand final clerkship grade distributions during the EM clerkship at a single medical school, focusing on differences between students who identified as URiM and those who identified as non‐URiM. We also investigated the contributions of components of the final clerkship grade to these differences including clinical evaluations and the National Board of Medical Examiners (NBME) shelf exam scores.

## METHODS

We used a cohort design consisting of 2 years of senior students who completed a required EM clerkship at a single accredited United States allopathic medical school. The study received exempt status from the institutional review board (HUM00226759).

This retrospective sample was drawn from University of Michigan Medical School students with graduation year (GY) 2021 or 2022. We used student self‐reported racial/ethnic identity as held by the medical school admissions office. We then categorized this variable as binomial variable of URiM or non‐URiM as determined by the AAMC definition: “Underrepresented in medicine means those racial and ethnic populations that are underrepresented in the medical profession relative to their numbers in the general population.”^23^

We looked for an association between identification as URiM versus non‐URiM and final clerkship grade. Overall clerkship grades were scored on a scale of honors, high pass, pass, and fail. These overall scores had three components. The first consisted of the National Board of Medical Examiners (NBME) shelf exam score, which accounted for 20% of the overall clerkship grade. Additionally, to be eligible for an “honors” grade, a student needed to score at least 80% on the NBME exam. The second component of the overall clerkship grade was the workplace‐based assessments accounting for 70%. These are standardized across the medical school and each evaluation is filled out on a 1–9 scale, with 1 being “poor: performance is consistently below expectations” and 9 being “exceptional: performance far exceeds expectations.” These assessments are completed by each supervising EM resident or faculty member on each assigned clinical shift. Third, there was a professionalism component, which accounted for 10% of the final grade that covers attendance and document/assignment completion. The rotation experience did not vary due to COVID‐19 pandemic precautions.

### Data analysis

Using a nonparametric Mann–Whitney *U*‐test, we compared the overall composite score on the EM clerkship, the EM NBME exam score, and clinical assessments between URiM‐ and non–URiM‐identifying students. Additionally, we also analyzed compared the projected clinical clerkship grade based on the workplace based assessments and professionalism scores alone after removing the NBME shelf exam score. Data analysis was completed with SPSS software (Version 28).

## RESULTS

**FIGURE 1:**
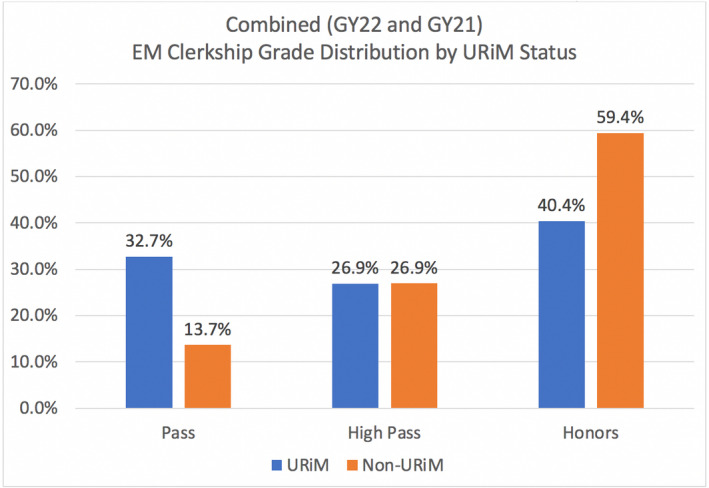
Grade data and breakdown by URiM status for overall, NBME subject exam, and clinical evaluations. GY, graduation year; NBME, National Board of Medical Examiners; URiM, underrepresented in medicine.

**FIGURE 2:**
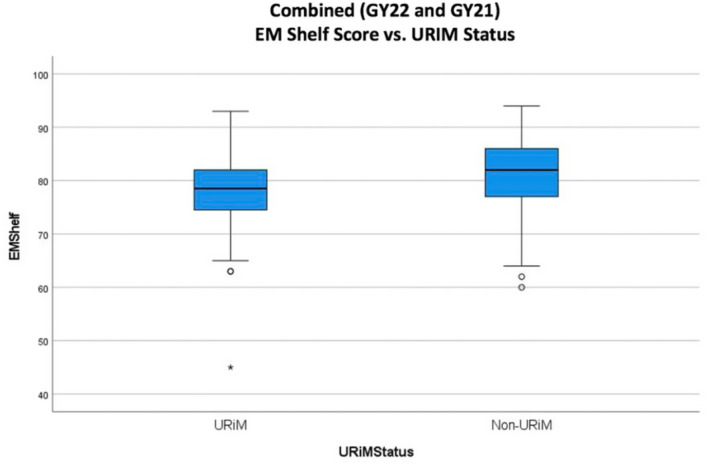
NBME shelf exam score by URiM versus non‐URiM status. GY, graduation year; NBME, National Board of Medical Examiners; URiM, underrepresented in medicine.

**FIGURE 3:**
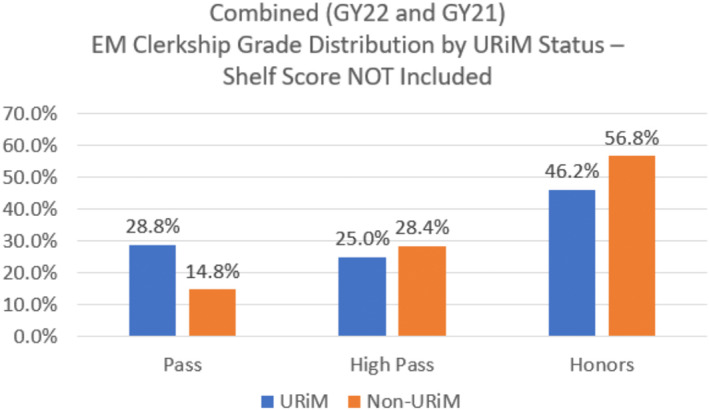
EM clerkship grade distribution by URiM status not including NBME shelf exam score. GY, graduation year; NBME, National Board of Medical Examiners; URiM, underrepresented in medicine.

A total of 334 students completed an EM rotation in GY 2021 and 2022. Fifty‐two (16.1%) identified as URiM while 271 (83.9%) identified as non‐URiM. Eleven students with “missing” race data were excluded, leaving a total population of 323. The non‐URiM group outperformed the URiM group on the NBME subject exam (81.2 vs. 77.6; *p* < 0.05). Additionally, the non‐URiM also outperformed the URiM cohort on clinical evaluations (6.71 vs. 6.49); however, there was no statistically significant difference between these scores (*p* = 0.057). Overall, clerkship grades differed with the URiM students having a higher percentage of “pass” grades (32.7%) and lower percentage of honors grades (40.4%) than non‐URiM students (13.7%, 59.4%; Figures 1, 2, 3).

Additionally, we attempted to control for the shelf exam score by removing this from the overall clinical clerkship grade and using the remaining 80% of the overall evaluation for the final grade. We found that there was still a difference between clinical performance between the URiM‐ and non–URiM‐identifying students, with more non‐URiM students achieving an honors grade (56.8%) than URiM‐identifying students (46.2%), though the discrepancy was smaller. There was still a higher number of URiM students receiving pass grades compared to their non–URiM‐identifying cohort (28.8% vs. 14.8%). Lastly, there were a higher number of “high pass” grades in the non‐URiM cohort than the URiM cohort (28.4%, 25.0%).

## DISCUSSION

In a single‐institution study, we found that our analysis of EM clerkship grades supports existing literature that there are differences in grade assignments when assessed by self‐reported URiM status.^3^, ^4^, ^5^, ^6^ Interestingly, there was no significant difference between URiM‐ and non–URiM‐identifying students with respect to clinical performance based on workplace‐based assessments. Initially, this suggested that the grading differences seen between the URiM‐ and non–URiM‐identifying students was largely driven by the NBME shelf exam scores. However, when corrected for NBME score, our data suggest that there are still differences in EM clerkship final grades between URiM and non‐URiM students. So, while there may not be a *statistically* significant difference with respect to the more subjective, workplace evaluations, our data suggest that these still may play a role in this outcome differential.

Hanson et al.^9^ elicited perspectives as to why these differences in overall clerkship grading outcomes exist in undergraduate medical education. These themes included the impact of systemic racism such as opportunity differentials for exposure and engagement with mentors; differences in commonality between student and evaluators; and increased potentials for bias, between URiM and non‐URiM students. Additionally, they suggest that microaggressions in the learning environment can affect student comfort and ultimately performance. Additionally, Teherani et al.^24^ suggest that even small differences in clerkship director evaluations of URiM compared to non‐URiM students contribute to a large difference in overall clerkship grades. These findings support our study results that while there were no statistically significant differences in workplace assessments, these small differences may play a larger functional role.

Our data raise questions on how we ameliorate these grading differentials. We explored removing the shelf exam contribution from our cohort, which would parallel the change to the USMLE Step 1 examination which recently moved to pass/fail scoring to potentially mitigate racial differences.^25^ This adjustment closed the racial gap on students who received an honors grade on the EM clerkship. However, it resulted in a higher percentage of non‐URiM students receiving high pass grades and a lower percentage of non‐URiM students receiving a pass grade. So overall racial differences in grading did not disappear. There is limited literature on the impact this change has had on performance differences for the high‐stakes USMLE exam. Our data suggest, however, that merely eliminating standardized testing will not ameliorate the differences seen in clinical grading.

How do we then move forward creating a more equitable grading and assessment system in undergraduate medical education if merely removing the standardized testing component is inadequate? Others have suggested transitioning entirely to a pass/fail system. Bullock et al.^26^ describe their transition from a tiered grading system (honors/high pass/pass) to a pass/fail curriculum. Overall, there was a sense from students that grading was more transparent and fair. It also allowed students to feel assessment was more mastery‐oriented than performance approach or performance avoid‐oriented. While more transparent and fair, data showed no difference in student perception on bias and presence of stereotype threat in grading evaluations, which further questions whether this is the best system to adopt to ensure an equitable assessment system. Jones et al.^25^ call for structural change in grading initiative by incorporating a holistic approach to clerkship evaluations rather than solely relying on standardized testing and clinical assessments. This approach, which parallels application review recommendations, may provide one path forward toward equity.^20^

Another path may involve the use of clear standardized, behaviorally anchored rubrics and trained grading committees. Colson et al.^6^ explored the impact of these interventions at a single institution. As a part of this comprehensive plan, grading committees also engaged in antibias training and the contribution to the final grade from subject matter exams was reduced. Finally, the overall grading scale changed to focus on competencies rather than a performance rating. The research team recently released a follow‐up publication describing the successes of this curricular change. Their initial analyses showed that students had a deep understanding of social determinants of health, bias, and how these have played a role in the field of medicine. Additionally, their students demonstrated competency to work with diverse patient populations and to directly address barriers to care. While no data have been shared yet from this program regarding grading disparities between URiM and non‐URiM cohorts with this assessment program change, its hope is to mitigate differences throughout the clinical medical education environment.^27^

Another approach that has the potential to both mitigate bias and provide a level of flexibility and catering to individualized learning is competency‐based medical education. Initially termed entrustable professional activities (EPAs), this is a framework that allows learners to demonstrate competency in key aspects of their professional life. Learners’ successes in these activities are assessed and graded on a scale of how much the supervisor would entrust a learner to perform an activity with independence.^28^, ^29^ Theoretically, this allows for more individualized education, as each individual learner has a unique set of skills, so some may be more initially entrustable in some aspects of training than others. This form of education allows learners to spend more time on targeted developmental domains and less time on activities that they have a higher level of mastery over. However, concerns remain that it may be difficult to truly assess all aspects and determine when a learner may be entrustable in a key aspect of a professional activity.^30^ Observable professional activities (OPAs) may serve as a helpful method to break down EPAs into smaller skill sets that can be more easily assessed. Once learners obtain comfort and skill in a set of OPAs, the combination of assessments in OPAs can determine entrustability in larger overall EPAs.^31^

By transitioning focus from generalized assessments to more behaviorally based and observable methods of assessments, grading and assessment can potentially become more equitable. Caretta‐Weyer and Gisondi^32^ established guidelines on how best to apply this new method of assessment into the clinical sphere, specifically in the emergency department. Key aspects of implementation require clearly defined learning goals and objectives for all aspects of the curriculum. These learning objectives would need to be directly observed to determine accurate assessments. Lastly, there would need to be a way to easily obtain and collect data to trend learner competencies over time to further delineate deficiencies in expertise and spend more individualized, focused time dedicated to progressing toward entrustability. By making more objective goals, assessments, and individualized approaches to learning, this could mitigate bias by focusing on clear, objective goals rather than more overarching assessments and impressions left by learners.

We know that racial disparities are prevalent throughout our health care system, and one way that has been shown to ameliorate these disparities is to increase the diversity in the workplace.^1^, ^2^ Studies demonstrate that in general, non–URiM‐identifying students receive lower scores on clerkship grades and less frequent induction into honors societies and score more poorly on standardized tests than their URiM colleagues.^3^, ^4^, ^5^, ^6^, ^7^, ^8^ This can have a direct impact on success in the residency match, thereby limiting the improvement for diversity in the health care field.^11^ EM is not exempt from this. As our study shows, there are discrepancies in clerkship grading between URiM and non‐URiM students, with respect both to standardized testing and to a potential, though not statistically significant, difference in workplace performance assessments. It is imperative to find a more inclusive, holistic approach to clerkship grading and assessment to mitigate these racial disparities.

## LIMITATIONS

This was a single‐center study, so further data and research will need to be done to ensure generalizability of this study. Additionally, in our cohort, there was a relatively small number of URiM students. This means that small changes in this cohort may have had a large impact on overall outcomes and statistics. One other drawback is that the racial data used in this study was student self‐reported data at time of matriculation to medical school. This reflects the student's identity in the application process which may be disclosed differently in various circumstance and may also differ from an external observer's assumptions. We also acknowledge that each medical school has a unique grading system, and the shelf exam and clinical evaluations may be weighted differently, making potential differences in final grade by racial identity more or less significant depending on each unique grading system. Lastly, due to limited sample size, we grouped all URiM‐identifying students in one group. Additional studies could further investigate differences by specific racial and ethnic identities.

## CONCLUSIONS

In this single‐center study, there were found to be grading differences between students who identified as underrepresented in medicine versus those who identified as non–underrepresented in medicine on their emergency medicine clerkship final grade. There is a statistically significant difference between these two groups with respect to outcomes on National Board of Medical Examiners shelf exam scores, which is responsible for a portion of the difference in these outcomes. However, when controlled for the standardized test scores, there was still a difference between these two groups. This calls for a change in how students are evaluated to address equity concerns in clinical assessments.

## CONFLICT OF INTEREST STATEMENT

The authors declare no conflicts of interest.
